# Distinct profiles of oxylipid mediators in liver, lung, and placenta after maternal nano-TiO_2_ nanoparticle inhalation exposure†

**DOI:** 10.1039/d2va00300g

**Published:** 2023-03-15

**Authors:** Todd R. Harris, Julie A. Griffith, Colleen E. C. Clarke, Krista L. Garner, Elizabeth C. Bowdridge, Evan DeVallance, Kevin J. Engles, Thomas P. Batchelor, William T. Goldsmith, Kim Wix, Timothy R. Nurkiewicz, Amy A. Rand

**Affiliations:** a Department of Chemistry and Institute of Biochemistry, Carleton University Ottawa ON K1S5B6 Canada Todd.Harris@carleton.ca; b Department of Physiology and Pharmacology, West Virginia University School of Medicine Morgantown WV 26506 USA; c Center for Inhalation Toxicology, West Virginia University School of Medicine Morgantown WV USA

## Abstract

Nano-titanium dioxide (nano-TiO_2_) is a widely used nanomaterial found in several industrial and consumer products, including surface coatings, paints, sunscreens and cosmetics, among others. Studies have linked gestational exposure to nano-TiO_2_ with negative maternal and fetal health outcomes. For example, maternal pulmonary exposure to nano-TiO_2_ during gestation has been associated not only with maternal, but also fetal microvascular dysfunction in a rat model. One mediator of this altered vascular reactivity and inflammation is oxylipid signaling. Oxylipids are formed from dietary lipids through several enzyme-controlled pathways as well as through oxidation by reactive oxygen species. Oxylipids have been linked to control of vascular tone, inflammation, pain and other physiological and disease processes. In this study, we use a sensitive UPLC-MS/MS based analysis to probe the global oxylipid response in liver, lung, and placenta of pregnant rats exposed to nano-TiO_2_ aerosols. Each organ presented distinct patterns in oxylipid signaling, as assessed by principal component and hierarchical clustering heatmap analysis. In general, pro-inflammatory mediators, such as 5-hydroxyeicosatetraenoic acid (1.6 fold change) were elevated in the liver, while in the lung, anti-inflammatory and pro-resolving mediators such as 17-hydroxy docosahexaenoic acid (1.4 fold change) were elevated. In the placenta the levels of oxylipid mediators were generally decreased, both inflammatory (*e.g.* PGE_2_, 0.52 fold change) and anti-inflammatory (*e.g.* Leukotriene B4, 0.49 fold change). This study, the first to quantitate the levels of these oxylipids simultaneously after nano-TiO_2_ exposure, shows the complex interplay of pro- and anti-inflammatory mediators from multiple lipid classes and highlights the limitations of monitoring the levels of oxylipid mediators in isolation.

## Abstract 2

A LC-MS/MS based platform is used to gain a global perspective on shifts in oxylipids, signaling molecules derived from dietary lipids that modulate the inflammatory response, after nano-TiO_2_ inhalation exposure.

## Graphical abstract

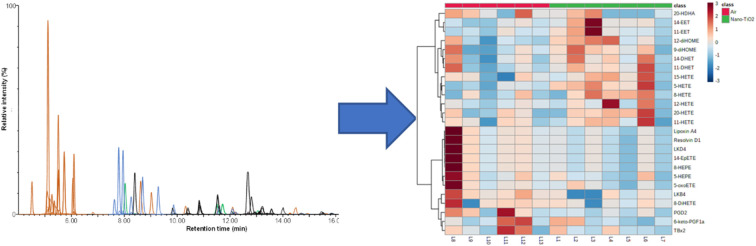

**Environmental significance**

Nano-titanium dioxide (nano-TiO_2_) is used in many industrial and consumer products, from paint to sunscreen. Recently, studies have linked gestational exposure to negative foetal and maternal health outcomes through the disruption of inflammatory pathways. Oxidized lipids (oxylipids) play a central role in the regulation of inflammation. Because hundreds of lipids are formed from dietary precursors *via* multiple enzymatic pathways, most studies focus on individual oxylipids. We use LC-MS/MS profiling to analyse global changes in oxylipid pathways after gestational exposure to nano-TiO_2_. We find that multiple oxylipids are perturbed by nano-TiO_2_ in the lung, liver, and placenta. This complex interplay of pro- and anti-inflammatory mediators from multiple lipid classes highlights the limitations of monitoring the levels of oxylipids in isolation.

## Introduction

Nano-titanium dioxide (nano-TiO_2_) is one of the most widely used engineered nanomaterials (ENMs) due to physiochemical properties such as high-refraction index, photocatalytic properties, and surface to mass ratio.^1^ Nano-TiO_2_ is utilized in a variety of products, ranging from paints, surface coatings, plastics, sunscreens, cosmetics,^1,2^ photoelectrochemical (PEC) biosensing applications, titanium implant coatings, and local drug delivery systems.^3^ Considering the variety of uses for ENMs, like nano-TiO_2_, the possibility of exposure can occur not only to workers in an occupational setting, but to consumers and patients as well during the lifespan of ENM enabled products. The wide use of ENMs has led to several studies investigating their potential toxic effects. In rats, pulmonary inflammation occurs after exposure to nano-TiO_2_ (ref. 4 and 5) *via* whole-body inhalation or instillation.^6,7^ Maternal nano-TiO_2_ inhalation during gestation results in maternal and fetal microvascular dysfunction in the uterine artery, fetal tail arteries, and placenta.^8,9^ It has also been associated with cognitive deficits in male offspring.^10^ Considered together, these studies reflect that systemic biologic effects follow inhalation exposure to nanomaterials and warrant further investigation.

Maternal nano-TiO_2_ inhalation exposure during gestation resulted in significantly decreased circulating TXB_2_, the stable thromboxane metabolite, in dam serum.^11^ Maternal nano-TiO_2_ inhalation exposed lungs had significantly increased gene expression of prostacyclin synthase (PGIS) and significantly decreased prostacyclin receptor gene expression.^11^ Serum from exposed dams also had increased 6-keto-PGF1α, the stable prostacyclin metabolite.^11^ Due to the increased mRNA expression of PGIS and elevated PGI_2_ serum levels, is likely in response to inflammatory signals arising from the lungs due to toxicant deposition within the lungs. Maternal nano-TiO_2_ inhalation exposure has been shown to increase interleukin (IL)-1β, 4, 5, and 13 in the plasma and bronchoalveolar lavage fluid.^12^ This indicates an innate inflammatory response, in which IL-1β has been shown to increase PGI_2_ production in hypoxic environments.^13^ The changes in both cyclooxygenase metabolites, led us to question the oxylipid modifications that may be occurring in response to the inflammatory signals spilling over from the lungs and acting on sites of toxicant removal (liver) and placental impacts from the inflammatory response to toxicants.

**Fig. 1:**
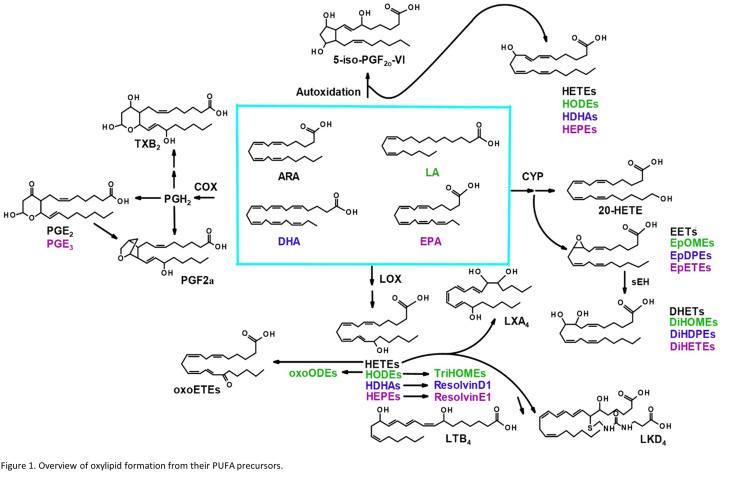
Overview of oxylipid formation from their PUFA precursors.

Oxylipins are produced from dietary lipids such as the omega-6 arachidonic (ARA) and linoleic acids and the omega-3 docosahexaenoic (DHA) and eicosapentaenoic (EPA) acids through enzymatic catalysis, as well as autooxidation in the presence of reactive oxygen species^14^ (Fig. 1). The oxylipid-producing enzymes form three major routes of metabolism, commonly called the cyclooxygenase (COX), the lipoxygenase (LOX) and cytochrome P450 pathways.^14^ Each parent dietary lipid is used to produce hundreds of potential lipid mediators *via* these pathways. For example, ARA is metabolized to prostaglandins by the COX pathway, leukotrienes by the LOX pathway, and epoxyeicosatrienoic acids (EETs) by the cytochrome P450 pathway.^14^

Oxylipids play a central role in human health and disease, modulating inflammation, vascular tone and pain, among other processes.^14^ Leukotrienes and prostaglandins have been shown to influence systemic responses after nanoparticle exposure *via* the lung. Silver nanoparticles upregulate the COX pathway and a prostaglandin, PGE_2_, in mice.^15^ Lead oxide exposure has been linked to an increase in F2 isoprostanes, autooxidation products of ARA, also in mice.^16^ Nano-TiO_2_ exposure has previously been shown to alter COX-mediated vasoreactivity in a rat model.^17^ Leukotriene B4 is elevated after human exposure to welding-derived nanoparticles.^18^

These studies examined a single or small numbers of oxylipids after pulmonary nanoparticle exposure. This targeted approach provides no insight into global changes that may be occurring in the lipidome. Normal biologic functions are maintained in part by the action of multiple oxylipid mediators, some with opposite physiological effects (*i.e.* certain oxylipids may have pro-inflammatory properties while others produced in the same pathway have anti-inflammatory and pro-resolving properties).^19,20^ An imbalance in these mediators typically results in dysfunction or disease. Therefore, when studying the role of these lipid mediators in toxicologic assessments, an ‘omics’ approach is crucial. Only by simultaneously assessing multiple oxylipids representative of each enzymatic pathway and parent lipid can we begin to understand how their combined effect impacts an organism's response to xenobiotic exposure.

Although there has been great interest in ARA metabolites in the field of nanoparticle and TiO_2_ exposure,^21,22^ no study has analyzed all three branches of the ARA cascade. Further, the metabolites formed from other omega-6 and omega-3 dietary lipids from nano-TiO_2_ inhalation exposure have not been explored. In this study, we take such an approach, using a sensitive UPLC-MS/MS based analysis to probe the oxylipid response in the liver, lung, and placenta of pregnant rats after inhalation exposure to nano-TiO_2_. We hypothesize that we will detect significant shifts in both anti- and pro-inflammatory mediators of biologic responses produced *via* enzymatic catabolism, alongside the autooxidation products formed by reactive oxygen species following nanomaterial inhalation exposures.

## Experimental

### Materials

Oxylipin standards were obtained from Cayman Chemical (Ann Arbor, MI). All lipid substrates were dissolved in methanol and combined to form stock solution mixes (1 μg mL^−1^) and stored under nitrogen at −20 °C until use. Water was either 18 mQ, or Omnisolv LC-MS grade (MilliporeSigma, Oakville, ON, Canada). HPLC and HiPerSolve LC-MS grade methanol and acetonitrile were purchased from VWR (Radnor, PA). Nano-TiO_2_ powder was obtained from Evonik (P25 Aeroxide TiO_2_, Parsippany, NJ) and was composed of a mixture of anatase (80%) and rutile (20%) TiO_2_. Particle characteristics have previously been determined, including primary particle size (21 nm), specific surface area (48.08 m^2^ g^−1^), and zeta potential (−56.6 mV).^23,24^

### Animal model

Female Sprague-Dawley (SD) rats were purchased pregnant from Hilltop Laboratories (Scottdale, PA) and housed in an American Association for Accreditation of Laboratory Animal Care (AAALAC) approved facility at West Virginia University (WVU). All animal procedures were performed in accordance with the Guidelines for Care and Use of Laboratory Animals of West Virginia University (WVU) and approved by the Animal Care and Use Committee of WVU. Rats were maintained under a regulated temperature of 20–26 °C, relative humidity of 30–70%, and a 12 : 12 hours light-dark cycle. Once acclimated for 48–72 hours, rats were randomly assigned to either air or nano-TiO_2_ exposure groups before mating. Rats had ad libitum access to food and water throughout the acclimation periods. All procedures were approved by the WVU Institutional Animal Care and Use Committee. To increase the likelihood of a viable pregnancy and retention of pups, pregnant rats were exposed after implantation was complete on gestational day (GD) 10–19 and exposed for a total of six days. On GD20, rats were weighed and then anesthetized with isoflurane gas (5% induction, 2–3.5% maintenance). Animals were placed on a warm heating pad and maintained at a rectal temperature of 37 °C. Rats were euthanized, and maternal liver and lung, along with placenta samples were collected. Collected tissues were flash frozen in liquid nitrogen, and stored in −80 °C.

### Inhalation exposure and aerosol characterization

Nano-TiO_2_ aerosols were generated as previously described.^21^ The output from a high-pressure acoustical generator (HPAG, IEStechno, Morgantown, WV) was fed into a Venturi pump (JS-60M, Vaccon, Medway, MA). The pump further de-agglomerates particles, which then enter the whole-body exposure chamber. The aerosol mass concentration in the exposure chamber is sampled in real-time by a personal DataRAM (pDR-1500; Thermo Environmental Instruments Inc, Franklin, MA). Acoustic energy is adjusted by feedback loops to maintain a stable mass aerosol concentration throughout the exposure. Calibration factors were obtained by gravimetric aerosol sampling measurements from Teflon filters and the DataRAM measurements. Each exposure had gravimetric measurements taken to calculate mass concentration measurements. Aerosol size distributions were measured in real-time in the exposure chamber at a target mass concentration of 12 mg m^−3^*via*: (1) a high-resolution electrical low-pressure impactor (ELPI+; Dekati, Tampere, Finland); (2) a scanning particle mobility sizer (SMPS 3938; TSI Inc., St. Paul, MN); (3) an aerodynamic particle sizer (APS 3321; TSI Inc., St. Paul, MN); and (4) a micro-orifice uniform deposit impactor (MOUDI 115R, MSP Corp, Shoreview, MN). Aerosol size distribution has previously been shown to have a count median diameter of 116 nm with a geometric standard deviation of 2.11.^20^ Bedding material was soaked to maintain proper humidity (20–70%). Air animals were exposed to HEPA-filtered air only, with similar temperature and humidity conditions within an exposure chamber that was used only for air exposed animals. Inhalation exposures were performed for 6 nonconsecutive days from GD 10–19 to maximize the chance of healthy gestations. Pregnant rats were exposed to a target concentration of 12 mg m^−3^, which was used previously for late gestation inhalation exposure studies.^24–26^ To estimate lung deposition (dose) with nano-TiO_2_ aerosols, the equation *D* = *FVCT* was used^27^ where *F* is the deposition fraction (10%), *V* is the minute ventilation (208.3 cc), *C* is mass concentration (mg m^−3^), and *T* equals the exposure duration (minutes).^23^ The exposure paradigm (12 mg m^−3^, 6 hours exposure, 6 days) produced a calculated cumulative lung deposition of 525 ± 16 μg with the last exposure occurring 24 hours prior to GD19. The calculations represent total lung deposition and do not account for lung clearance (MPPD Software v 2.11, Arlington, VA).

### Oxylipin tissue extraction

Liver, placenta, and lung tissues were sent to Carleton University on dry ice and stored at −20 °C until extraction. Approximately 180 mg of each tissue was weighed into 2 mL tubes containing 1.4 mm ceramic beads designed for liver and placenta soft tissue homogenization or 2.8 mm ceramic beads designed for lung hard tissue homogenization (Bertin Corp., Rockville, MD). To the vials, a 1 μg mL^−1^ labelled internal standard stock solution in methanol (2.5 μL) was added, along with ice-cold methanol containing 0.1% acetic acid and 0.1% BHT (400 μL) and an antioxidant solution containing 0.2 mg mL^−1^ of BHT and EDTA (10 μL). Each tissue sample was homogenized using a Bertin Minilys tissue homogenizer for 20 seconds with a 30 second rest on ice in between, and repeated 3 times for liver and placenta, and 4 times for lung tissue. Samples were then centrifuged at 13 200 rpm for 10 minutes. The methanol supernatant was transferred to 2 mL polypropylene centrifuge vials and stored at −20 °C overnight. Methanol suspensions were subsequently diluted with 1.6 mL 18 mQ water so that the solvent ratio was 80 : 20 water : methanol, prior to loading onto SPE cartridges. Oasis HLB cartridges (3 mL, 60 mg, 30 μm) (Waters, Milford, MA) were used for oxylipin extraction and clean-up from tissue matrices. Cartridges were each washed with 1 column volume of HPLC grade methanol and 2 volumes of HPLC grade ethyl acetate, and then conditioned with 2 volumes of 80 : 20 water : methanol. Samples were loaded and then washed with 2 volumes of 80 : 20 water : methanol. Cartridges were dried by vacuum for 10 minutes (15 mmHg). Analytes were then eluted with 0.5 mL methanol and 2 mL ethyl acetate into 2 mL centrifuge tubes containing 5 μL of trapping solution (30% glycerol in methanol). The eluates were dried by vacuum using an Eppendorf Vacufuge (Hamburg, Germany) at 30 °C and reconstituted in 50 μL LC-MS grade methanol. All samples were mixed on a vortex and spin-filtered (0.1 μm PVDF, MilliporeSigma, St. Louis, MO) at 13 200 rpm for 10 minutes at 10 °C prior to being transferred to 2 mL LC-MS amber vials containing a 100 μL glass insert. Extracts were stored at −20 °C until analysis by UPLC-MS/MS.

### LC-MS/MS analysis

All samples were run using a Waters Acquity UPLC I-Class PLUS system coupled to a Waters Xevo TQS-Micro triple quadrupole mass spectrometer. Lipids were separated using a Phenomenex Kinetex C18 column (150 × 2.1 mm, 1.7 μm, 100 Å) (Torrance, CA) warmed to 40 °C. Injection volume was 5 μL. The LC mobile phases were 95 : 5 water : acetonitrile (A) and acetonitrile (B) both containing 0.1% formic acid. Gradient separation conditions were as follows: initial conditions of 90 : 10 A : B holding for 0.5 minutes (*t* = 0.5 minutes), decreasing to 20 : 80 A : B over 4.5 minutes (*t* = 5 minutes), decreasing to 5 : 95 A : B over 0.1 minutes (*t* = 5.1 minutes), holding for 2.9 minutes (*t* = 8 minutes), reverting to initial conditions over 2 minutes (*t* = 10 minutes). The solvent flow rate was 0.3 mL min^−1^. The MS/MS analysis was performed in ESI negative mode. The capillary voltage was set at 2.5 kV, with a desolvation temperature and flow of 300 °C and 500 L per hour, respectively. The cone flow was 150 L per hour, and the source temperature was 150 °C. The nebulizer gas was set at 6 bar. All analytes were monitored using the multiple reaction monitoring (MRM) mode as precursor and product ion mass transition pairs. Dwell time was automated, the length depending on how many transitions were in each channel (number of transitions ranged from 10–30). Points-per-peak was set at 12. Analytes were identified and quantified based on their LC retention time and one MRM transition. The relative response ratios of each analyte compared to their respective internal standard were used to calculate concentrations. A detailed list of the 68 oxylipins monitored in this study (including 12 labelled internal standards), their precursor and product ions, the optimized cone and collision energies, and LC-retention times can be found in Table S1.† A representative chromatograph depicting each oxylipin MRM transition is presented in Fig. S1.†

### QA/QC procedures

Linearity, sensitivity (LOD and LOQ), and absolute recovery was assessed prior to sample analysis. Analyte linearity was calculated using serial dilutions (by factors of 2, 4, 8, 16, and 32) of an intrastudy QC that contained 5 μL of all extracted samples. For those oxylipins below the LOD in this QC sample, linearity was instead calculated using from the external calibration curve of its pure standard in methanol. Linearities for each oxylipin were acceptable, all having *R*^2^ values ≥0.98. The instrumental LOD and LOQ were determined using either the standard deviation of the response of an external calibration curve, or the standard deviation arising from the peak-to-peak noise of the baseline to the signal of an analytical peak in a standard in methanol, using a minimum signal-to-noise-ratio of 3 and 10 respectively.^28^ Linearity and sensitivity values can be found in Table S2.† Absolute recovery of the labelled oxylipin internal standards was calculated and performed in duplicate. Recovery values ranged from 71.2–95.5%. Standard deviations were <30% (except for d^11^-11-EET and d^4^-12,13-diHOME). Absolute recovery values are found in Table S3.† Results were in the range of an oxylipin validation study,^29^ which reported absolute recoveries from 45–84% using a similar procedure. Prior to injecting samples, a system suitability QC was used to ensure the instrument met its threshold performance.^30^ It contained a 50 ng mL^−1^ mixture of 8 representative oxylipins standards in methanol. To pass the suitability test, shifts in retention times were <2%, peak intensity was ±10% of a predefined area, and had no evidence of peak splitting. During sample analysis, methanol blanks were run after every five samples to assess analyte carryover. No carryover was observed. Finally, sample extracts from the same tissue were run in one batch to avoid inter-day variability in analyte response. Individual treatments (*e.g.*, air *vs.* nano-TiO_2_ exposed tissues) were mixed when setting up the sample run list to minimize analysis bias.

### Statistical analysis

Normality test (Shapiro–Wilk), equal variance test (Brown–Forsythe), Student's *t*-test and Mann–Whitney rank sum test were performed in SigmaPlot v14 (Systat, Inc., Palo Alto, CA) to establish significance. Principal component analysis (PCA) and hierarchical cluster analysis was performed in MetaboAnalyst 5.0.^31^ Data were mean-centered and divided by the standard deviation of each variable. Missing values were replaced by 1/5 of the minimum positive values of their corresponding variables.

## Results and discussion

Nano-TiO_2_ inhalation exposure caused a shift in inflammatory oxylipids in the lung, reflecting an impact on the COX branch of the ARA cascade. Changes in the levels of oxylipids produced in one branch of the cascade often affect the levels of oxylipids produced by other branches, as well as metabolites derived from dietary lipids that can metabolized by the COX, LOX, and CYP enzymes.^32,33^ These dietary lipid include linoleic acid, DHA and EPA, parent molecules to hundreds of oxylipids, many of which have bioactive properties. Differential profiles in these oxylipids are one measure of the state of inflammation of an organ. Oxylipids have both inflammatory and anti-inflammatory properties, and their balance gives an indication of how or if an organ's inflammatory state has changed, as well as whether it is in the process of resolution or in the early stages of an inflammatory response. Our LC-MS/MS based platform currently quantitates the levels of 56 oxylipids which are representative of three branches of the ARA cascade alongside select autooxidation products and metabolites of other omega-6, and omega-3 dietary lipids. In this study we first used the platform to gain a global perspective of the oxylipid shifts in the lung, where increased inflammation can lead to structural remodelling of the airway.^34^ The observed impact of nano-TiO_2_ on the lung oxylipid profile, as well as the earlier observation that nano-TiO_2_ inhalation exposure caused elevated serum levels of inflammatory interleukins, raised the possibility of inflammatory effects in other organs. We next examined the liver, a key site of toxicant removal, where inflammation is directly related to conditions such as fibrosis.^35^ Placental tissue was available from the previous study. Oxylipids have been shown to play a central role in placental health and disease.^36–38^ For this reason we used our oxylipid platform to quantitate the lipids after nano-TiO_2_ inhalation exposure. Placental inflammation can impact both fetal retention and fetal development, as well as play a role in preeclampsia, a hypertensive condition that can affect the health of both mother and fetus.^39,40^

We performed unbiased targeted lipidomic profiling using tandem quadrupole mass spectrometry (MS/MS) with ultra-performance liquid chromatography (UPLC) on 10 rats exposed to nano-TiO_2_ nanoparticles *via* inhalation matched with 9 air-control rats during gestation. Rats were dissected to remove the lung (*n* = 10 nano-TiO_2_, *n* = 9 air), liver (*n* = 7 nano-TiO_2_, *n* = 6 air), and placenta (*n* = 10 nano-TiO_2_, *n* = 8 air) tissues. A total of 56 oxylipins were screened in each tissue, 25 derived from ARA, 7 from LA, 12 from DHA, and 12 from EPA. As depicted in Fig. 1, oxylipins targeted in this study were formed though the cyclooxygenases (COX), lipoxygenases (LOX), or cytochrome P450s (CYP) enzymatic pathways or autooxidation of their parent omega-6 or omega-3 polyunsaturated fatty acid.

**Fig. 2:**
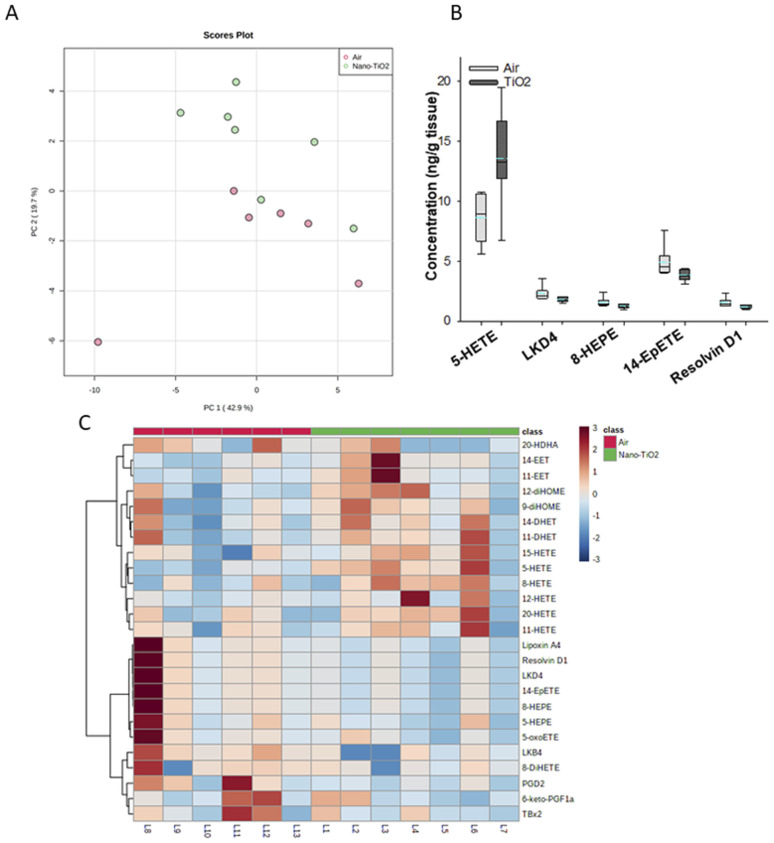
Analysis shows altered lipidome in air *vs.* nano-TiO_2_ liver tissue. (A) Principal component analysis. (B) Box plots of significantly altered oxylipids. (C) Heat map visualization of the top 25 discriminating features between air and nano-TiO_2_ treatments. For panel B, the boundary of the box indicates the 25th and 75th percentile. The horizontal line in the box in the median value, and the whiskers are the 10th and 90th percentiles. The dashed horizontal blue line is the mean value (all outliers represented by filled circles). *N* = 6 for air group, *N* = 7 for nano-TiO_2_ group. Oxylipid concentrations are reported in Table S4.†

Principal component analysis of the oxylipids in the liver tissue revealed a moderate separation, with 60% of the variation in the data accounted for by the first two principal components (Fig. 2A). As a screening criterion, we examined lipids with a *p*-value less than or equal to 0.05 (Fig. 2B). This yielded five lipids total, two derived from ARA, 5-HETE and LKD4. 5-HETE, elevated in the treated group (FC = 1.6), is a known pro-inflammatory oxylipid. Produced from ARA by 5-LOX, 5-HETE has been shown to be a hepatic inflammatory mediator in several liver disease models, including non-alcoholic fatty liver disease (NAFLD) and a genetic model of atherosclerosis.^41,42^ LKD4 (FC = 0.8), also produced in the LOX branch, has been shown to constrict pre- and post-sinusoidal veins in an isolated rat liver.^43^ Three omega-3 metabolites were identified, two derived from EPA, 8-HEPE (FC = 0.8) and 14-EpETE (FC = 0.8), and one derived from DHA, Resolvin D1 (FC = 0.8). Resolvin D1 has been shown to have a direct anti-inflammatory effect in a murine hepatic fibrosis model.^44^ HEPEs have been found to be elevated in lung tissue after exposure to diesel exhaust and urban air particles, but studies have not directly linked them to liver inflammation.^45^ An EpETE isomer has recently been shown to reduce inflammation in *in vitro* and *in vivo* lung models.^46^

We next performed hierarchical clustering heatmap analysis (Fig. 2C) to investigate changes in the top 25 metabolites responsible for the observed variation. Interestingly, the ARA-derived HETEs, as well as the ARA products in the P450 branch were elevated in the treatment group (with the exception of 20-HDHA), while the products in the LOX and COX branch, including oxylipids displaying inflammatory and oxylipids displaying anti-inflammatory properties, were lowered. Of the elevated lipids, the HETEs, of which 5-HETE is a member, are of particular interest. The HETEs are a class of enzymatically and non-enzymatically produced oxylipids with a general inflammatory effect in the liver and other tissues.^14^ In contrast, the general anti-inflammatory products of DHA and EPA were lowered in the treated group.

In the liver, pro-inflammatory lipid mediators derived from ARA were elevated in the treated rats, in particular the HETEs. In addition, both pro- and anti-inflammatory metabolites in the COX-2 and LOX pathways were lowered in the treated group, as well as anti-inflammatory lipids derived from EPA and DHA, indicating that the liver is in the pro-inflammatory stage of its response to nano-TiO_2_ inhalation exposure.

**Fig. 3:**
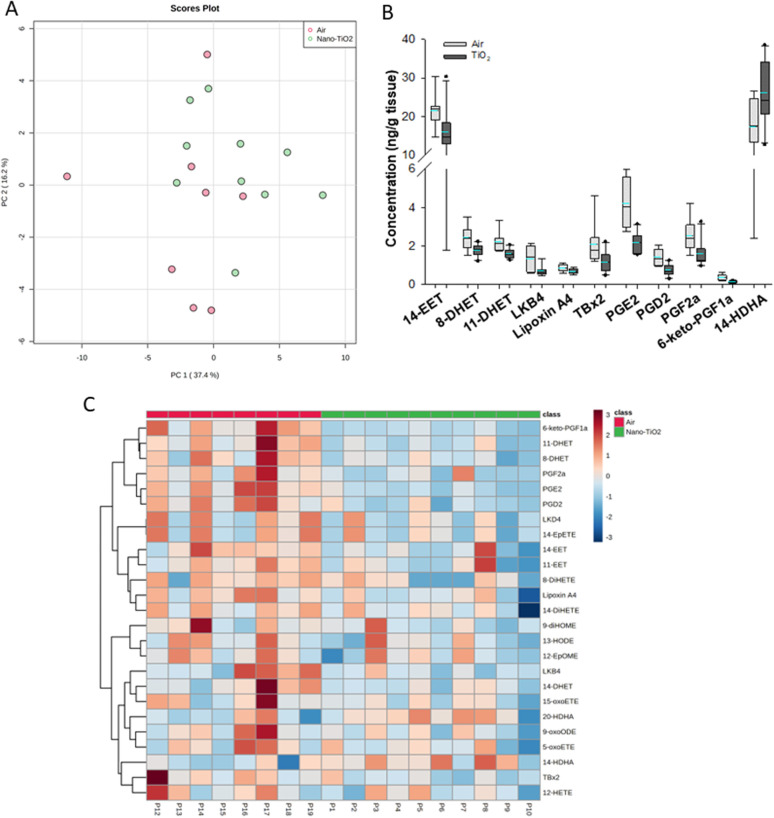
Analysis shows altered lipidome in air *vs.* nano-TiO_2_ placental tissue. (A) Principal component analysis. (B) Box plots of significantly altered oxylipids. (C) Heat map visualization of the top 25 discriminating features between air and nano-TiO_2_ treatments. For panel B, the boundary of the box indicates the 25th and 75th percentile. The horizontal line in the box in the median value, and the whiskers are the 10th and 90th percentiles. The dashed horizontal blue line is the mean value (all outliers represented by filled circles). *N* = 9 for Air group, *N* = 10 for nano-TiO_2_ group. Oxylipid concentrations are reported in Table S6.†

The same statistical analysis was employed for the lung oxylipids. PCA resulted in a moderate separation between the control and treated groups (Fig. 3A). Applying our criteria, a *p*-value less than or equal to 0.05, we identified five oxylipids (Fig. 3B). There were significant changes in two ARA-derived lipids, 11-EET and 6-keto-PGF1a. 11-EET was increased in the nano-TiO_2_ group (FC = 1.2). Treatment with 11-EET and boosting the levels of 11-EET using inhibitors of its primary route of metabolism has been shown to reduce lung inflammation caused by LPS treatment and ischemia/reperfusion injury, respectively.^47,48^ 6-keto-PGF1a, decreased in the treated group (FC = 0.6), is a marker of prostacyclin, a potent vasodilator in pulmonary vasculature.^49^ In addition to these ARA metabolites, two pro-resolving metabolites of DHA were significantly increased in the lungs of the treated mice, 4-HDHA (FC = 1.5) and 20-HDHA (FC = 1.4). The HDHA have been implicated in many lung inflammatory models, including a murine silver nanoparticle-induced acute inflammation model.^50,51^ While the 4- and 20-HDHA isomers were not mentioned in these studies, 20-HDHA has been shown to reduce the levels of inflammatory cytokines in a neuronal cell culture study.^52^ 4-HDHA is a marker for D series resolvin pathway, lipids that have been shown to have a pro-resolving activity.^53^ Finally, one DiHETE, a lipid derived from EPA, was identified as decreased, 11-DiHETE (FC = 0.2). EPA is epoxidized by P450s to form EpETEs, which are then hydrolyzed by soluble epoxide hydrolase to form the DiHETEs (Fig. 1). The EpETE are anti-inflammatory mediators, 17-EpETE reducing inflammation in TNF-α-pretreated human bronchi.^54^ Less is known about the bioactivity of DiHETE in the lung, but relevant to this study, 5,15-DiHETE was correlated with increased eosinophilia in lungs of neonates born to mice exposed to urban air particles.^45^

Because no significant changes in the corresponding EpETE (ESI material Table S1†) were detected, this increase in DiHETEs may indicate an upregulation or increased activity of soluble epoxide hydrolase, perhaps accompanied by concomitant upregulation or increase in activity of the P450s involved in epoxidation of EPA. Overall, these data support that after nano-TiO_2_ inhalation exposure, the inflammatory response in the lung had entered a pro-resolving or anti-inflammatory stage, the levels of anti-inflammatory oxylipid mediators increased and the pro-inflammatory oxylipid mediators decreased.

Hierarchical clustering heatmap analysis supports this picture (Fig. 3C), with exceptions. In general, anti-inflammatory lipids derived from EPA (HEPE), DHA (HDHAs), ARA (EETs, Lipoxin A4) and linoleic acid (9-oxoODE) were increased in the treated group while inflammatory mediators derived from linoleic acid (EpOMEs) and ARA (PGD2), were decreased, along with some anti-inflammatory mediators.

**Fig. 4:**
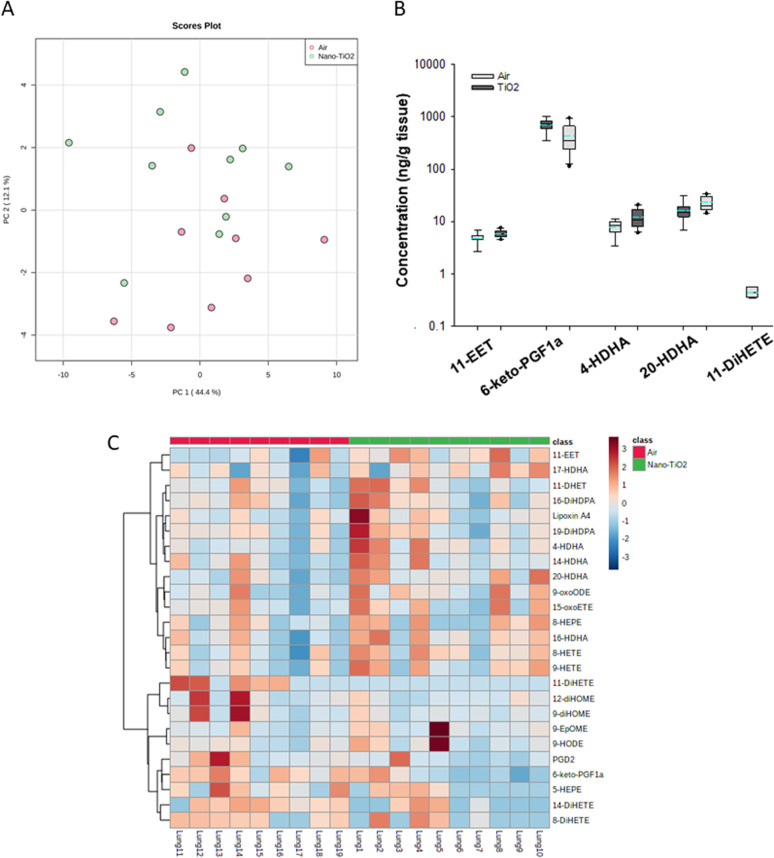
Analysis shows altered lipidome in air *vs.* nano-TiO_2_ lung tissue. (A) Principal component analysis. (B) Box plots of significantly altered oxylipids. (C) Heat map visualization of the top 25 discriminating features between air and nano-TiO_2_ treatments. For panel B, the boundary of the box indicates the 25th and 75th percentile. The horizontal line in the box in the median value, and the whiskers are the 10th and 90th percentiles. The dashed horizontal blue line is the mean value (all outliers represented by filled circles). *N* = 9 for air group, *N* = 10 for nano-TiO_2_ group. Oxylipid concentrations are reported in Table S5.†

When we performed PCA of the placenta data, we found a moderate separation of the treated and untreated groups (Fig. 4A). Again, using a screening criteria of a *p*-value less than or equal to 0.05, we identified eleven lipids (Fig. 4B). Ten metabolites of ARA were significantly decreased in the treated group. 14-EET (FC = 0.7), 8-DHET (FC = 0.7) and 11-DHET (FC = 0.7) are metabolites in the P450 branch of the ARA cascade. The DHET are produced from the EETs through the hydrolytic action of sEH.^32^ The EETs are increased in preeclamptic human placentas compared with normal pregnancy,^37^ a disease with a strong inflammatory component.^55^ Two metabolites in the LOX branch were significantly decreased, LKB4 (FC = 0.5) and lipoxin A4 (FC = 0.7). LKB4 has been proposed as a potential blood marker of preeclampsia,^56^ while lipoxin A4 has been found to be decreased in the placenta during preeclampsia in a rat model.^57^ Five lipids in the COX branch were also significantly decreased, TXB2 (FC = 0.6), PGE2 (FC = 0.5), PGD2 (FC = 0.5), PGF2a (FC = 0.6), and 6-keto-PGF1a (FC = 0.4). Thromboxane is increased in human preeclampsia placentas while prostacyclin, of which 6-keto-PGF1a is a marker, is decreased.^58^ The prostaglandins PGE2, PGD2, and PGF2a have been shown to play a central role in normal pregnancy in several mammalian models.^36,38,59^ In placental tissues, 14-HDHA, a product of DHA autooxidation, increased rather than decreased in the treated rats (FC = 1.5). 14-HDHA is a maresin pathway marker that has been identified in maternal and umbilical cord blood of mothers given omega-3 supplements in early and late pregnancy.^60^ Maresin is a pro-resolving lipid mediator.^61^

It was noted that all of the lipids identified in the placenta except 14-HDHA were decreased after treatment. Hierarchical clustering heatmap analysis confirmed that most oxylipids in the three branches of the ARA signaling cascade were decreased in the nano-TiO_2_ treated group, as well as oxylipids derived from linoleic acids and EPA (Fig. 4C). The shift cannot be described as being anti-inflammatory or inflammatory, since both pro-inflammatory oxylipids (such as thromboxane A4 and PGE2) and anti-inflammatory oxylipids (such as lipoxin A4) were simultaneously decreased.

## Conclusions

In conclusion, nano-TiO_2_ inhalation exposure results in systemic biologic response characterized by oxylipid signaling in the liver, lung, and placenta. In the liver we found the challenge elevated pro-inflammatory mediators, while in the placenta, both anti-inflammatory and pro-resolving mediators were increased. In the placenta levels of lipid signaling mediators were reduced across the board, with some exceptions. This study shows the limitations of focusing on a single oxylipid mediator, whether inflammatory (*e.g.*, PGE2) or anti-inflammatory (*e.g.*, LXB4), as these mediators work in concert with dozens of other oxylipids, creating a complex balance that shifts from organ to organ, as well as temporally as the inflammatory response progresses. A single oxylipid mediator has its effect as one node in a network of pro- and anti-inflammatory oxylipids, whose levels are affected by diet, something that must be considered when making inferences regarding human health.

Our results also raise several questions for future studies, two of which will be discussed here. First, what implications do our findings have on questions of diet and toxicity. The omega-3 omega-6 balance, in particular, has been studied in many areas of health and disease. Here we have found that in the liver and lung, anti-inflammatory mediators derived from both lipid classes were significantly raised. In the placenta, lipid signaling in general was decreased. A study modulating diet could determine if the inflammatory response could be altered in these organs by altering the ratios of parent lipids. It would be particularly interesting to see if this downregulation in lipid signaling in the placenta is maintained as the omega-6 : omega-3 ratio is shifted. Second, how are these oxylipids regulated? There are three ways that these shifts in lipid metabolism can occur. First, by modulating the expression or activity of the involved enzymes. Second, by shifting the levels of their substrates, the free fatty acids, by increasing/decreasing the esterification of the oxylipids to the plasma membrane or increasing/decreasing their release. Third, a combination of both processes might occur. A multi-omic approach looking at the proteome and transcriptome alongside the lipidome will give a clearer picture of how this modulation in lipids is achieved.

## Conflicts of interest

There are no conflicts to declare.

## Supplementary Material

VA-002-D2VA00300G-s001
