# Supplementary material for: Distinct profiles of oxylipid mediators in liver, lung, and placenta after maternal nano-TiO2 nanoparticle inhalation exposure
Source: Env Sci Adv. 2023 Mar 15;2(5):740–8. doi: 10.1039/d2va00300g (PMC10167894; doi:10.1039/d2va00300g)
Supplement: VA-002-D2VA00300G-s001 [file VA-002-D2VA00300G-s001.pdf]

Electronic Supplementary Material (ESI) for Environmental Science: Advances.
This journal is © The Royal Society of Chemistry 2023

Supplementary Materials

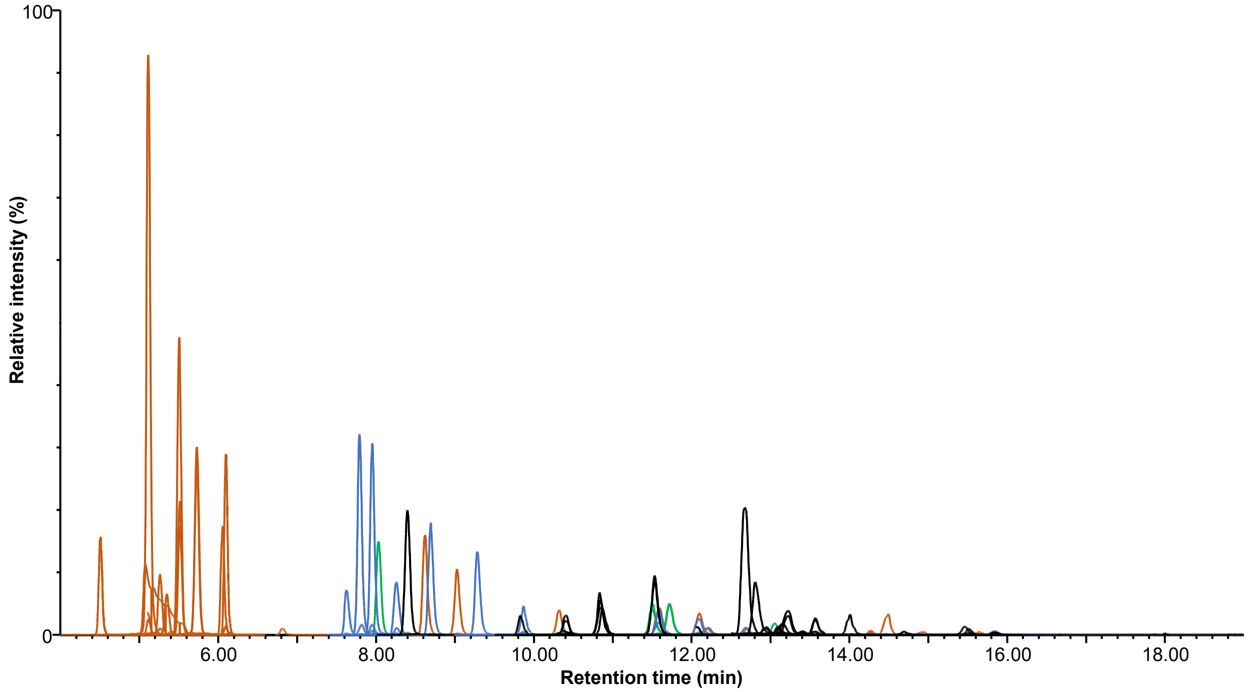

Figure S1. Representative LC-MS/MS chromatograph of a 100 ng/mL standard containing 56 oxylipins. Signals were acquired using multiple reaction monitoring (MRM) operating in ESI negative mode.

Table S1. Oxylipins monitored in this study. The table contains 68 compounds (including 12 internal standards), their precursor and product ions, the optimized cone and collision energies, as well as retention time and the respective internal standard for matrix effect correction.

Analyte	Internal Standard	Precursor Ion (m/z)	Product Ion (m/z)	Cone (V)	CE (V)	RT (min)
PGE_2	d14-PGE_2	351.4	271.5	30.0	15.0	5.0
d4-PGE_2	-	355.5	275.4	10.0	15.0	5.0
PGD_2	d14-PGE_2	351.2	271.3	20.0	15.0	5.2
d4-$PGF_{2\alpha}$	-	357.3	197.3	55.0	25.0	4.8
$PGF_{2\alpha}$	d14-PGE_2	353.4	193.3	70.0	25.0	4.8
6-keto-$PGF1_{\alpha}$	d14-PGE_2	369.4	163.4	50.0	25.0	4.1
TBX2	d4-TBX2	369.1	168.9	10.0	15.0	4.6
d4-TBX2	-	373.3	199.3	20.0	15.0	4.6
8,9-EET	d11-11,12-EET	319.5	155.1	50.0	10.0	15.9
11,12-EET	d11-11,12-EET	319.1	167.2	40.0	15.0	15.3
d11-11,12-EET	-	330.2	167.1	30.0	10.0	15.3
14,15-EET	d11-11,12-EET	319.4	219.3	30.0	10.0	14.8
8,9-DHET	d11-14,15-DHET	337.3	127.2	20.0	20.0	9.4
11,12-DHET	d11-14,15-DHET	337.4	167.4	25.0	15.0	8.8
14,15-DHET	d11-14,15-DHET	337.4	207.2	25.0	15.0	8.2
d11-14,15-DHET	-	348.5	207.4	25.0	15.0	8.1
5-Iso-$PGF_{2\alpha}$-VI	d4-PGF2a	353.0	115.0	50.0	20.0	4.8
5-HETE	d8-15-HETE	319.3	257.5	40.0	10.0	14.0
8-HETE	d8-15-HETE	319.2	155.2	30.0	10.0	13.6
9-HETE	d8-15-HETE	319.3	151.3	20.0	15.0	13.3
11-HETE	d8-15-HETE	319.2	167.2	20.0	15.0	13.2
12-HETE	d8-15-HETE	319.2	179.2	25.0	20.0	12.8
15-HETE	d8-15-HETE	319.4	219.2	10.0	10.0	12.2
d8-15-HETE	-	327.3	182.6	15.0	15.0	12.2
20-HETE	d6-20-HETE	319.3	275.3	50.0	15.0	10.4
d6-20-HETE	-	325.3	281.4	35.0	15.0	10.4
5-oxoETE	d8-15-HETE	317.7	203.2	50.0	20.0	15.5
15-oxoETE	d8-15-HETE	317.2	113.1	20.0	15.0	12.9

Lipoxin A4	d4-LKB4	351.2	115.2	30.0	15.0	5.6
LKB4	d4-LKB4	335.2	195.3	40.0	15.0	7.3
d4-LKB4	-	339.4	197.2	40.0	15.0	7.3
LXD4	d4-LKB4	495.3	177.3	60.0	20.0	5.5
9,10-EpOME	d4-9,10-EpOME	295.3	171.1	55.0	15.0	14.5
d4-9,10-EpOME	-	299.2	172.3	60.0	15.0	14.4
12,13-EpOME	d4-9,10-EpOME	295.3	195.2	45.0	15.0	14.2
9,10-DiHOME	d4-12,13-diHOME	313.3	201.2	30.0	20.0	7.9
12,13-DiHOME	d4-12,13-diHOME	313.3	183.3	60.0	20.0	7.5
d4-12,13-DiHOME	-	317.1	185.3	40.0	20.0	7.5
9-HODE	d4-9-HODE	295.3	171.3	30.0	15.0	11.3
d4-9-HODE	-	299.2	172.3	35.0	15.0	11.2
13-HODE	d4-9-HODE	295.3	195.2	50.0	15.0	11.1
9-oxoODE	d3-9-oxoODE	293.4	185.2	40.0	20.0	12.7
d3-9-oxoODE	-	296.3	296.5	50.0	20.0	12.5
7,8-DPE	d11-11,12-EET	343.1	189.3	15.0	10.0	14.6
16,17-DPE	d11-11,12-EET	343.2	233.2	20.0	10.0	13.9
19,20-DPE	d11-11,12-EET	343.3	281.3	25.0	10.0	13.4
7,8-DiHDPA	d11-14,15-DHET	361.2	113.3	40.0	15.0	9.8
16,17-DiHDPA	d11-14,15-DHET	361.2	233.2	40.0	15.0	8.6
19,20-DiHDPA	d11-14,15-DHET	361.4	273.4	25.0	15.0	8.2
4-HDHA	d8-15-HETE	343.0	101.1	20.0	15.0	14.5
14-HDHA	d8-15-HETE	343.3	281.5	10.0	10.0	12.8
16-HDHA	d8-15-HETE	343.5	233.3	15.0	10.0	12.2
17-HDHA	d8-15-HETE	343.4	281.3	20.0	10.0	11.9
20-HDHA	d6-20-HETE	343.0	240.9	20.0	10.0	11.7
Resolvin D1	d4-LKB4	375.0	141.0	20.0	15.0	5.5
PGE3	d14-PGE_2	349.0	269.0	20.0	15.0	4.6
8,9-EpETE	d11-11,12-EET	317.2	255.4	45.0	10.0	13.5
11,12-EpETE	d11-11,12-EET	317.3	167.4	15.0	10.0	13.2
14,15-EpETE	d11-11,12-EET	319.4	219.3	30.0	10.0	13.0
8,9-DiHETE	d11-14,15-DHET	335.2	127.1	30.0	20.0	7.7
11,12-DiHETE	d11-14,15-DHET	335.2	167.1	30.0	15.0	7.4
14,15-DiHETE	d11-14,15-DHET	335.3	207.2	30.0	15.0	7.3

5-HEPE	d8-15-HETE	317.3	5.5	10.0	10.0	11.6
8-HEPE	d8-15-HETE	317.4	155.2	30.0	10.0	10.9
12-HEPE	d8-15-HETE	317.2	179.3	10.0	10.0	11.0
15-HEPE	d8-15-HETE	317.2	219.4	10.0	10.0	10.5
18-HEPE	d6-20-HETE	317.0	215.0	20.0	10.0	9.9

Table S2. Analyte validation parameters, including linearity, sensitivity (LOD/LOQ) as expressed in vial concentration (ng/mL).

Analyte	Linearity (R^2)	LOD (ng/mL)	LOQ (ng/mL)
PGE2	0.997	0.008	0.027
PGD2	0.984	0.010	0.035
PGF2A	0.994	0.031	0.105
TXB2	0.996	0.034	0.114
8,9-EET	0.988	23.8**	35.4**
11,12-EET	0.987	3.41	11.4
14,15-EET	0.997*	0.350	1.16
8,9-EET	0.988	0.510	1.70
11,12-DHET	0.985	0.019	0.062
8,9-DHET	0.999	0.022	0.075
14,15-DHET	0.989	0.183	0.609
5-Iso-PGF2a-VI	0.997	10.9**	33.0**
5-HETE	0.991	0.060	0.201
8-HETE	0.977	0.049	0.163
9-HETE	0.995	14.5**	43.9**
11-HETE	0.973	0.063	0.209
12-HETE	0.996	0.925	3.09
15-HETE	0.994	0.077	0.257
20-HETE	0.999	0.909	3.03
5-OxoETE	0.992	24.4**	73.8**
15-OxoETE	0.985	20.0**	60.6**
6-keto-PGF1a	0.991	19.2**	58.3**
Lipoxin A4	0.997*	0.018	0.060
LKB4	0.976	0.180	0.600
LKD4	0.990*	1.03	3.34

9,10-EpOME	0.986	0.219	0.729
12,13-EpOME	0.993	0.435	1.45
9,10-DiHOME	0.993	0.075	0.250
12,13-DiHOME	0.997	0.073	0.242
9-HODE	0.996	0.047	0.156
13-HODE	0.986	0.04	0.135
9-OxoODE	0.987	0.130	0.434
7,8-DPE	0.945*	0.684	2.28
16,17-DPE	0.990*	0.390	1.30
19,20-DPE	0.942*	0.123	0.411
7,8-DiHDPE	0.999*	0.125	0.416
16,17-DiHDPE	0.998*	0.025	0.084
19,20-DiHDPE	0.993	0.091	0.302
4-HDHA	0.991*	33.5**	102**
14-HDHA	0.985	0.065	0.217
16-HDHA	0.994	0.029	0.097
17-HDHA	0.996*	0.094	0.313
20-HDHA	0.989	15.4**	46.7**
Resolvin D1	0.996*	10.1**	30.7**
PGE3	0.990*	16.1	53.5
8,9-EpETE	0.996*	185	618
11,12-EpETE	0.992*	108	361
14,15-EpETE	0.995*	80.6	269
8,9-DiHETE	0.997*	39.3	131
11,12-DiHETE	0.998*	14.1	47.1
14,15-DiHETE	0.999*	13.8	46.0
5-HEPE	0.976	13.2	43.9
8-HEPE	0.994*	20.5	68.5
12-HEPE	0.988	163	545
15-HEPE	0.996*	37.9	126
18-HEPE	0.999	12.4**	37.5**

*Linearity was calculated using oxylipin standards in methanol rather than the endogenous oxylipin response from the intrastudy control, since these lipids fell below the LOD in the sample.
**The LODs and LOQs were calculated based on the standard deviation of a standard curve of pure oxylipins in methanol.

Table S3. Absolute percent recovery calculated using the mass labelled oxylipin internal standards. Recovery and standard deviations were obtained in duplicate analysis in a liver and placental matrix, comparing the LC-MS/MS peak areas of samples spiked in internal standards prior to homogenization and extraction vs. a sample spiked after extraction.

Analyte	Recovery ± Std Dev (%)
d14-PGE_2	72.7 ± 7.6
d4-PGF2a	83.2 ± 9.7
d4-TXB_2	68.8 ± 9.9
d11-11-EET	79.4 ± 42.3
d8-14,DHET	83.6 ± 11.3
d8-15-HETE	71.2 ± 16.7
d6-20-HETE	74.3 ± 26.1
d4-LKB4	95.5 ± 30.3
d4-9,10-EpOME	80.9 ± 19.3
d4-12,13-diHOME	95.5 ± 42.5
d4-9-HODE	73.7 ± 25.1
d3-9-oxoODE	69.9 ± 20.8

Table S4. Oxylipid concentrations in liver tissues (ng/gram tissue).

	L1	L2	L3	L4	L5	L6	L7	L8	L9	L10	L11	L12	L13
	TiO_2	TiO_2	TiO_2	TiO_2	TiO_2	TiO_2	TiO_2	Air	Air	Air	Air	Air	Air
9-oxoODE	9.56	14.04	12.87	9.59	6.52	9.99	5.68	12.24	8.5	6.99	11.95	7.45	6.79
9-EpOME	60.46	60.71	60.52	55.85	44.75	60.2	25.43	69.65	53.2	18.03	71.11	46.53	41.91
9-HODE	53.33	60.76	65.08	63.91	49.63	62.53	33.45	59.91	51.31	16.64	76.46	42.27	51.89
13-HODE	45.5	46.07	56.29	50.59	37.88	47.22	25.82	44.75	47.71	17.77	58.92	36.19	43.39
12-EpOME	42.48	39.97	40.25	39.62	28.97	37.42	18.43	39.5	37.97	13.78	45.2	31.38	33.56
12-diHOME	59.9	68.48	76.16	79.82	46.05	54.02	36.16	67.65	41.69	24.45	46.91	42.31	38.15
9-diHOME	17.17	25.45	19.18	17.67	14.8	19.7	8.48	24.64	7.44	6.95	12.6	16.75	13.51

15-oxoETE	NA	0.65	NA	NA	0.95	0.95	0.77	NA	0.9	NA	NA	0.96	0.35
12-HEPE	0.45	0.4	0.46	0.64	0.33	NA	0.37	0.81	NA	0.43	NA	0.82	0.68
5-oxoETE	2.21	2.63	2.28	1.96	1.64	2.29	1.81	3.96	2.49	2.11	2.35	2.4	2.17
5-HEPE	3.29	2.85	2.92	2.51	2.33	3.61	2.31	5.05	3.43	2.7	3	3.53	2.89
8-HEPE	1.36	1.21	1.4	1.21	1.01	1.41	1.11	2.43	1.53	1.3	1.44	1.47	1.33
14-EpETE	4.21	3.75	4.35	3.74	3.13	4.36	3.45	7.53	4.74	4.03	4.47	4.56	4.13
8-HETE	NA	2.28	3.95	2.78	3	3.8	0.76	NA	2.13	NA	1.08	2.77	0.6
11-EET	11.28	17.87	32.01	9.67	7.43	7.57	3.8	4.68	6.48	1.92	10.6	6.96	6.94
11-HETE	54.26	77.24	89.83	89.51	60.79	125.75	28.28	78.35	69.53	24.16	79.94	74.17	43.65
12-HETE	4.56	9.11	6.12	22.69	4.08	15.86	1.11	7.24	4.62	2.09	5.86	7.92	6.15
9-HETE	2.7	4.81	4.34	4.38	4.35	6.5	4.68	4.84	5.57	2.58	5.26	5.3	2.65
5-HETE	13.3	14.26	16.68	12.79	11.91	19.48	6.7	7.06	8.58	5.61	10.77	10.57	9.28
20-HETE	0.18	14.23	13.54	17.42	16.09	27.56	NA	15.12	0.2	2.54	14.51	10.83	NA
15-HETE	13.57	16.88	22.57	23.83	17.02	29.95	9.08	18.28	16.86	5.97	NA	19.74	14.7
14-EET	11.1	23.73	45.41	12.76	12.83	14.33	4.49	8.65	1.87	1.59	11.45	14	7.73
11-DiHETE	NA	1.3	NA	1.25	1	1.48	1.01	2.32	NA	1.19	NA	1.42	1.29
LKB4	0.8	NA	NA	0.87	0.59	0.82	0.65	1.42	0.9	0.76	0.85	1.09	0.78
8-DiHETE	1.93	1.72	NA	1.71	1.44	2	1.58	3.46	NA	1.85	2.05	2.09	1.89
14-DiHETE	0.71	0.8	0.77	0.8	0.55	0.9	0.7	1.19	0.65	0.57	0.53	0.72	0.69
8-DHET	4.18	4.77	4.3	4.01	3.37	5.29	3.13	6	3.34	2.7	3.83	3.94	2.63
11-DHET	5.76	7.78	6.16	6.58	5.61	10.04	3.76	9.46	3.64	2.67	4.27	5.74	4.28
14-DHET	6.54	9.57	6.36	7.42	5.56	9.46	4.52	8.88	3.91	2.54	5.82	6.69	4.23
7-DPE	4.93	4.4	5.09	4.37	3.66	5.11	4.04	8.82	5.56	4.71	5.24	NA	4.83
20-HDHA	1.62	2.56	3.27	NA	NA	NA	1.32	2.9	2.41	1.55	NA	3.69	1.59
14-HDHA	NA	11.49	3.67	16.84	2.64	28.73	NA	21.89	NA	6.21	3.78	3.85	7.96
16-HDHA	3.96	4.93	4.97	3.75	3.01	6.42	2.45	5.91	4.49	3.39	4.27	4.66	3.16
PGD2	3.54	3.99	1.9	4.61	2.45	5.35	1.39	10.61	7.55	0.9	15.46	6.25	2.95
Lipoxin A4	0.95	0.85	0.98	0.88	0.75	0.98	0.78	1.7	1.07	0.91	1.01	1.03	0.93
PGE2	4.65	4.71	3.03	5.72	4.1	7.85	2.27	9.51	2.96	1.41	9.58	7.05	4.02
PGF2a	8.04	10.37	4.2	13.81	7.19	16.12	3.78	21.28	5.33	NA	17.62	10.5	10.31

16-DiHDPA	3.59	3.68	4.01	5.48	3.49	6.72	2.63	7.67	3.39	2.02	3.39	4.31	2.77
7-DiHDPA	2.74	2.38	2.44	2.1	1.76	2.45	1.94	4.58	2.67	2.26	2.52	2.56	NA
19-DiHDPA	2.25	2.12	2.23	1.92	1.64	2.18	1.17	2.67	1.75	1.24	1.93	1.92	1.55
TBx2	2.38	1.23	0.97	2.2	1.05	1.03	0.71	2.13	1.44	0.64	4.02	3.22	0.34
Resolvin D1	1.33	1.18	1.37	1.18	0.98	1.37	1.09	2.37	1.49	1.27	1.41	1.44	1.3
LKD4	1.97	1.76	2.04	1.75	1.47	2.04	1.62	3.53	2.22	1.89	2.1	2.14	1.93
6-keto-PGF1a	1.13	1.09	0.32	0.47	0.18	NA	0.44	0.66	0.23	0.47	1.53	1.68	0.39

Table S5. Oxylipid concentrations in lung tissues (ng/gram tissue).

Sample	Lung1	Lung2	Lung3	Lung4	Lung5	Lung6	Lung7	Lung8	Lung9	Lung10	Lung11	Lung12	Lung13	Lung14	Lung15	Lung16	Lung17	Lung18	Lung19
Treatment	TiO2	TiO2	TiO2	TiO2	TiO2	TiO2	TiO2	TiO2	TiO2	TiO2	Air	Air	Air	Air	Air	Air	Air	Air	Air
9-oxoODE	102.84	62.03	83.95	70.88	70.19	62.08	49.75	101.59	62.9	87.79	61.23	68.2	58.17	99.86	50.17	50.78	38.24	65.32	55.88
9-EpOME	371.22	379.12	200.89	212.14	873.19	263.8	189.45	232.6	245.76	292.23	247.69	243.38	214.86	435.18	209.02	180.32	124.24	242.31	223.66
9-HODE	418.94	328.29	163.72	260.21	742.1	191.05	130.55	230.44	184.81	243.84	273.28	248	280.53	289.57	177.49	169.95	122.13	276.74	241.8
13-HODE	687.11	531.11	302.97	380.44	356.44	261.96	196.05	340.23	339.06	506.42	502.65	493.68	602.71	563.09	297.09	292.42	217.73	523.64	426.52
12-EpOME	485.6	471.33	298.88	270.95	331.46	293.04	238.09	296.68	312.98	465.92	395.65	407.6	371.04	625.85	281.04	281.41	178.21	312.67	339.89
12-diHOME	32.02	21.88	13	14.46	20.08	19.13	19.56	18.54	28.58	21.97	19.66	59.33	15.93	64.41	24.74	14.56	15.13	20.62	23.49
9-diHOME	11.3	8.07	4.51	6.6	7.72	6.44	7.31	5.48	7.82	6.83	8	20.54	5.55	24.32	9.11	5.75	3.49	6.84	5.97
15-oxoETE	71.84	52.71	39.27	60.51	33	33.03	26.02	71.51	31.78	61.71	43.32	44.9	45.25	65.12	40.66	38.2	21.02	36.46	39.75
12-HEPE	18.9	13.85	5.8	17.6	11.41	6.18	4.55	NA	13.58	11.7	9.24	16.5	18.86	14.66	6.74	10.82	4.36	8.21	9.66
18-HEPE	7.95	5.66	4.51	NA	4.7	3.71	3.53	6.6	3.19	9.13	4.84	4.72	4.17	8.21	5.29	3.27	2.09	5.58	3.76
15-HEPE	14.33	8.55	4.54	NA	5.99	NA	4.65	7.2	9.17	9.38	7.72	10.88	10.36	9.98	6.28	3.98	2.2	8.51	4.67
5-oxoETE	82.49	56.14	37.35	71.83	36.4	42.02	29.11	49.75	44.59	46.18	53.49	32.28	69.21	57.42	36.44	44.19	22.73	56.03	49.07
5-HEPE	NA	2.58	2.72	2.95	1.91	NA	NA	NA	NA	NA	2.16	NA	4.55	2.61	1.12	2.07	1.29	NA	3.65
8-HEPE	3.2	2.96	NA	3.45	NA	NA	NA	3.02	2.06	3.42	2.34	NA	1.6	3.26	1.93	NA	0.98	1.3	NA
8-HETE	43.97	38.15	21.88	43.94	34.89	32.09	17.3	31.16	30.66	37.77	34.05	20.38	25.73	36.45	26.08	18.86	9.82	36.47	19.93
11-EET	5.76	5.31	7.11	6.46	4.58	5.45	5.84	7.63	4.54	6.26	4.63	4.61	4.48	5	5.82	4.75	2.67	6.9	4.62
11-HETE	415.87	379.18	210.22	382.2	240.11	278.12	186.77	283.13	262.1	293.73	400.5	267.23	380.39	430	228.9	239.37	121.1	308.67	236.47
12-HETE	298.24	341.09	135.43	328.83	180.29	195.1	103.77	156.56	272.64	334.77	199.31	317.04	296.04	294.36	139.25	214.56	70.79	252.16	172.96

9-HETE	171.01	141.07	46.24	144.31	81.3	68.08	51.67	117.85	114.73	146.3	115.06	61.45	91.1	117.15	72.66	51.22	28.14	111.69	48.86
5-HETE	217.96	148.63	167.18	189.94	97.96	97.06	64.2	121.49	112.23	148.89	116.77	74.94	278.74	159.66	84.37	114.12	62.8	146.2	175.42
20-HETE	43.74	46.26	40.3	22.38	27.89	31.45	23.59	75.16	27.89	56.25	42.79	30.23	66.88	46.46	24.68	59.33	12.07	28.15	29.82
15-HETE	1310.42	1179.13	534.14	1223.2	778.14	841.43	606.17	889.63	873.64	976.93	1054.43	741.87	996.03	1165.08	721.64	670.14	313.64	1056.14	608.59
14-EET	8.91	NA	7.07	7.7	4.67	4.49	3.24	5.86	4.65	7.2	5.39	5.09	5.41	5.51	5.41	5.74	3.07	8.31	4.23
8-EET	13.02	4.86	6.89	11.85	5.37	6.63	8.65	10.11	7.8	10.1	7.82	6.62	7.26	7.12	9.03	8.86	5	13.45	6.77
11-DiHETE	NA	NA	NA	NA	NA	NA	NA	0	0	0	0.57	0.54	NA	0.43	0.34	0.36	NA	NA	NA
LKB4	9.99	7.51	5.21	8.29	4.67	4.56	3.25	6.41	4.68	8.16	6.55	6.73	8.61	9.76	4.88	5.57	3.38	8.72	6.18
8-DiHETE	NA	1.34	NA	1.3	0.97	NA	0.52	NA	0	0	0.97	0.95	0.91	0.87	0.85	NA	0	0.74	0.86
14-DiHETE	NA	NA	0.62	0.89	0.78	NA	0.39	0	NA	0	NA	0.72	0.65	0.82	0.79	0.63	0.47	0.57	0.63
8-DHET	11.67	9.22	5.96	NA	6.43	4.57	3.52	6.36	4.3	6.02	7.76	6.03	5.04	9.94	7.84	9.17	3.17	4.76	3.97
11-DHET	20.14	20.34	13.31	18.72	10.84	8.39	6.59	11.84	8.41	9.94	11.51	10.75	10.31	17.88	12.76	12.24	5.39	9.44	6.58
14-DHET	22.42	20.08	13.27	18.98	10.38	9.04	7.07	13.84	9.96	13.8	12.95	11.5	13	19.39	13.91	19.38	7.22	13.6	7.9
4-HDHA	20.95	17.02	8.24	17.8	10.12	10.77	6.12	11.42	7.15	10.84	10.31	7.15	8.3	9.26	9.06	6.77	3.48	11.2	5.69
20-HDHA	32.71	29.65	17.56	18.41	20.38	19.81	15.7	29.28	14.5	34.03	16.26	17.45	13.43	30.88	20.65	15.17	6.79	14.94	11.32
14-HDHA	229.36	198.82	97.28	215.83	85.67	106.07	71.72	109.27	86.2	102.5	164.06	75.99	109.27	190.1	113.19	57.24	35.31	78.64	59.02
17-HDHA	9.41	NA	7.2	10.17	6.98	9.66	5.61	13.7	9.34	13.22	9.07	5.69	8.53	NA	8.38	6.32	NA	10.83	4.5
16-HDHA	39.93	46.64	18.94	41.86	25.97	29.01	17.56	33.61	30.26	36.57	37.17	21.98	27.38	39.06	25.16	16.95	8.04	23.7	17.23
PGD2	160.93	153.24	372.94	139.9	137.36	97.91	83.34	113.65	80.81	146.71	174.11	286.56	463.13	278.24	124.94	144.6	150.22	166.88	187.29
Lipoxin A4	1.54	0.85	0.6	0.88	0.77	0.43	0.33	0.57	0.65	0.7	0.73	0.58	0.51	0.78	0.51	0.48	0.28	0.76	0.58
PGE2	131.84	155.24	111.65	105.36	99.54	83.37	68.2	108.02	82.71	92.09	99.92	93.79	151.91	125.77	87.67	95.63	53.24	121.39	87.69
PGF2a	95.75	98.67	55.59	61.42	48.17	55.35	45.19	65.5	44.15	64.8	68.19	50.75	71.69	97.06	51.64	58.36	34.17	57.7	60.18
5-IPF2a-VI	NA	2.25	1.65	1.98	1.33	1.22	NA	NA	1.4	1.66	1.97	1.16	1.39	1.9	1.7	NA	NA	1.32	1.16
16-DiHDPA	3.77	3.48	2.44	2.51	2.24	1.82	1.11	2.81	1.47	2.33	2.16	2.33	2.09	3.06	2.75	2.05	0.98	1.74	1.39
19-DiHDPA	14.96	10.04	8.47	9.21	5.91	4.85	2.31	6.99	4.99	7.37	7.35	7.02	6.82	8.14	7.84	4.94	2.62	7.97	3.83
TBX2	82.81	90.96	126.07	57.27	44.42	81.63	43.27	72.39	54.81	81.77	48.63	44.28	151.98	73.68	77.61	111.05	27.97	31.04	62.82
6-keto-PGF1a	808.92	958.92	616.37	414.82	362.26	231.55	247.14	340.89	115.04	268.89	747.54	758.59	1027.6	694.43	349.41	847.07	679.15	523.49	832.98

Table S6. Oxylipid concentrations in placental tissues (ng/gram tissue).

	P1	P2	P3	P4	P5	P6	P7	P8	P9	P10	P12	P13	P14	P15	P16	P17	P18	P19
	TiO2	TiO2	TiO2	TiO2	TiO2	TiO2	TiO2	TiO2	TiO2	TiO2	Air	Air	Air	Air	Air	Air	Air	Air
9-oxoODE	11.48	9.90	13.22	10.39	12.72	8.21	13.50	11.04	9.37	6.90	10.08	12.16	13.25	10.07	16.49	18.97	11.42	10.46
9-EpOME	24.33	16.14	34.25	25.86	31.29	17.19	37.96	24.59	16.63	14.43	22.37	42.35	31.25	17.89	25.30	41.42	22.47	20.89
9-HODE	21.78	14.92	41.49	27.17	32.70	20.60	33.99	26.16	16.46	12.80	20.85	38.45	29.60	24.52	21.99	41.65	22.04	23.18
13-HODE	20.64	15.19	54.18	31.40	32.63	24.29	43.36	27.82	19.84	15.04	25.87	48.47	46.44	29.21	29.17	53.76	33.49	25.74
12-EpOME	NA	15.50	44.96	24.87	32.27	19.10	39.58	22.43	15.59	13.18	24.77	43.17	35.09	19.14	26.70	46.28	28.47	19.21
12-diHOME	3.28	3.32	6.50	2.81	3.15	1.84	4.41	2.08	1.94	2.96	2.20	5.65	7.81	2.57	1.98	5.67	3.19	3.33
9-diHOME	1.78	1.71	2.86	1.64	1.56	1.38	1.97	1.36	1.14	1.59	1.88	1.99	3.42	1.61	1.28	2.36	1.75	1.91
15-oxoETE	4.94	4.80	5.50	3.79	5.89	3.71	5.67	5.59	3.89	2.68	6.91	6.56	3.38	4.34	5.75	10.02	4.94	5.14
5-oxoETE	14.44	11.13	10.91	12.39	13.17	11.45	11.58	15.12	8.79	7.03	10.30	14.21	12.85	9.83	17.44	16.65	12.99	10.40
5-HEPE	NA	NA	1.77	1.62	2.00	1.86	NA	2.00	1.45	1.71	NA	1.66	2.32	1.80	1.64	NA	1.91	2.33
8-HEPE	0.80	1.08	0.82	0.78	0.96	0.96	0.84	0.96	0.70	0.82	1.13	0.80	1.12	0.83	NA	1.06	0.92	1.12
14-EpETE	2.46	3.36	2.55	2.42	2.99	2.77	2.32	2.98	2.16	2.55	3.49	2.48	3.46	2.58	2.45	3.28	2.85	3.48
8-HETE	6.37	5.87	8.75	6.61	8.60	5.56	6.07	9.73	4.97	4.21	4.95	6.85	6.94	5.20	5.22	8.51	6.34	4.91
11-EET	11.65	16.77	14.87	12.26	10.54	10.69	15.60	24.35	7.65	6.77	12.99	15.10	19.10	14.94	15.82	21.76	16.56	18.72
11-HETE	78.82	76.67	108.50	84.39	106.74	66.72	97.66	108.35	67.57	50.35	75.92	90.24	83.28	66.64	78.80	142.26	85.99	68.09
12-HETE	26.64	18.31	40.63	30.74	41.11	24.89	27.00	32.44	27.21	15.75	52.41	38.47	22.60	29.20	34.44	44.61	29.47	21.92
9-HETE	10.63	9.42	21.33	11.77	15.47	8.51	11.97	17.83	9.67	6.67	8.60	17.28	15.37	9.09	10.18	18.53	16.51	9.36
5-HETE	23.53	16.92	29.09	23.50	23.49	18.84	20.50	29.60	15.04	12.77	18.72	24.99	22.22	17.11	23.80	32.19	22.23	11.15
20-HETE	NA	0.14	0.11	NA	1.37	0.12	NA	NA	1.35	0.11	NA	NA	NA	1.76	0.10	0.14	NA	NA
15-HETE	62.20	53.93	76.73	59.85	90.65	48.54	64.63	85.56	50.42	35.36	59.29	60.10	45.03	46.15	63.77	117.07	64.84	56.25
14-EET	14.18	18.42	15.39	18.54	13.50	13.37	17.03	30.37	11.42	7.93	14.75	19.58	30.42	22.55	22.76	21.67	19.07	22.19
8-EET	6.05	9.18	7.13	7.40	10.19	6.89	13.89	15.35	1.94	3.62	3.30	8.33	8.02	12.03	12.45	18.40	8.27	2.98
11-DiHETE	0.69	0.94	0.71	0.68	NA	0.79	0.67	0.83	0.60	0.71	0.98	0.73	NA	NA	NA	0.92	0.80	0.97
LKB4	0.54	0.63	1.33	0.60	0.71	NA	0.78	NA	0.59	0.48	0.66	0.60	0.65	NA	2.13	2.01	1.42	1.93
8-DiHETE	1.13	1.54	1.17	1.11	NA	NA	NA	1.37	0.99	NA	1.60	NA	1.59	1.18	1.12	1.50	1.31	1.60
14-DiHETE	0.29	0.40	0.30	0.29	0.35	0.33	0.28	0.35	0.26	NA	0.41	0.29	0.41	0.31	0.29	0.39	0.34	0.41
8-DHET	1.59	2.04	1.98	1.72	2.25	1.84	1.79	2.02	1.23	1.45	2.43	1.53	2.98	2.27	1.83	3.51	2.52	2.41

11-DHET	1.45	1.82	1.60	1.43	1.78	1.50	1.63	2.08	1.28	1.32	2.05	1.77	2.41	1.73	1.89	3.31	2.21	2.39
14-DHET	0.68	0.72	0.68	0.66	0.89	0.43	0.93	0.95	0.77	0.47	0.79	0.80	0.46	0.85	0.95	1.94	1.13	1.31
4-HDHA	5.82	6.04	4.88	6.32	6.99	6.14	5.38	8.16	4.22	4.06	7.42	5.17	6.71	4.99	6.75	6.46	4.46	6.39
20-HDHA	2.93	4.08	4.05	4.47	6.10	3.79	5.87	5.74	3.79	NA	3.16	2.19	2.76	2.41	4.98	6.30	2.93	NA
14-HDHA	24.51	22.05	33.44	23.37	23.78	36.49	16.38	38.58	30.50	12.72	21.55	16.78	13.37	13.50	18.43	26.59	2.41	25.67
17-HDHA	NA	14.40	38.43	25.27	50.48	26.38	NA	30.79	21.11	NA	27.57	22.17	29.04	15.68	NA	21.11	NA	24.65
16-HDHA	3.28	NA	4.90	4.25	6.48	3.76	3.92	5.59	3.36	NA	5.42	4.05	NA	3.39	3.39	6.42	3.87	5.11
PGD2	1.25	0.76	0.55	0.65	1.27	0.32	0.91	0.71	0.62	0.53	1.54	1.02	1.81	0.96	1.87	2.07	1.18	0.95
Lipoxin A4	0.60	0.86	0.58	0.58	0.67	0.66	0.76	0.92	0.49	NA	0.94	0.60	0.91	0.77	1.09	1.10	0.69	0.79
PGE2	3.15	2.35	2.24	1.99	2.48	1.61	2.81	2.12	1.57	1.57	4.27	2.76	4.88	2.85	5.85	5.97	3.35	3.78
PGF2a	1.81	1.25	1.22	1.79	2.03	0.99	3.29	1.20	1.21	1.24	2.56	1.89	2.86	1.54	3.18	4.24	1.95	2.27
16-DiHDPA	0.16	0.20	0.16	0.08	0.26	0.17	0.20	0.24	0.18	0.08	0.11	0.20	0.11	0.21	0.20	0.34	0.22	0.11
7-DiHDPA	1.49	1.89	1.53	1.36	1.68	1.56	NA	1.68	1.22	1.43	1.96	NA	1.94	1.45	1.38	1.84	1.60	1.96
19-DiHDPA	0.69	0.90	0.77	NA	0.85	0.74	0.65	0.85	0.60	0.68	0.94	NA	0.97	0.68	0.74	0.89	NA	0.97
TBx2	2.25	0.96	1.32	1.22	1.57	0.74	0.50	1.55	0.66	1.07	4.61	1.55	2.02	1.28	2.52	2.15	1.23	1.52
Resolvin D1	0.78	1.06	0.80	0.76	0.99	0.87	0.73	0.94	0.68	0.80	1.10	NA	1.09	0.81	0.77	1.03	0.90	1.10
LKD4	1.15	1.57	1.19	1.13	1.40	1.30	1.09	1.40	1.01	1.19	1.64	1.16	1.62	1.21	1.15	1.54	1.34	1.63
6-keto-PGF1a	0.13	0.16	0.15	0.17	0.22	0.14	0.16	0.22	0.10	0.08	0.51	0.21	0.41	0.25	0.25	0.62	0.43	0.35
